# Predictors of Developmental and Respiratory Outcomes Among Preterm Infants With Bronchopulmonary Dysplasia

**DOI:** 10.3389/fped.2021.780518

**Published:** 2021-11-25

**Authors:** Iris Morag, Efrat Barkai, Yaara Wazana, Arnon Elizur, Orly Levkovitz Stern, Orna Staretz-Chacham, Shiran Pinchevski-Kadir, Noa Ofek Shlomai

**Affiliations:** ^1^The Edmond and Lily Safra Children Hospital, Shebe Medical Center, Ramat Gan, Israel; ^2^Sackler School of Medicine, Tel Aviv University, Tel Aviv, Israel; ^3^Faculty of Management, Tel Aviv University, Tel Aviv, Israel; ^4^Shamir Medical Center, Institute of Allergy, Immunology and Pediatric Pulmonology, Zerifin, Israel; ^5^Department of Neonatology, Meir Medical Center, Kfar Saba, Israel; ^6^Metabolic Clinic, Pediatric Division, Soroka Medical Center, Ben-Gurion University, Be'er Sheva, Israel; ^7^Department of Neonatology, Hadassah and Hebrew University Medical Center, Jerusalem, Israel

**Keywords:** development, outcomes, preterm infant, machine learning, bronchopulmonary dysplasia

## Abstract

**Objectives:** To examine the importance of perinatal and postnatal environmental factors on developmental and respiratory outcomes among preterm infants with bronchopulmonary dysplasia (BPD).

**Methods:** Preterm infants (<32 weeks of gestation) born at a single tertiary medical center between 2012 and 2015 were included. Development was assessed at 12 months corrected age. Parents retrospectively completed a health and lifestyle questionnaire reviewing their child's health during the first 2 years of life. A linear regression model was applied to assess the effect of various perinatal and postnatal factors on development. A machine-learning algorithm was trained to assess factors affecting inhaler use.

**Results:** Of 398 infants meeting the inclusion criteria, 208 qualified for the study: 152 (73.1%) with no BPD, 40 (19.2%) with mild BPD, and 16 (7.7%) with moderate-severe BPD. Those in the moderate-severe group were more likely to be male, have mothers who were less educated, and require longer ventilation periods and less time to regain birth weight. They were also more likely to have mothers with asthma/allergies and to have a parent who smoked. Those in the moderate-severe BPD group exhibited significantly lower developmental scores (85.2 ± 16.4) than the no-BPD group (99.3 ± 10.9) and the mild BPD group (97.8 ± 11.7, *p* < 0.008) as well as more frequent inhaler use (*p* = 0.0014) than those with no or mild BPD. In addition to perinatal factors, exposure to breast milk, income level and daycare attendance positively affected development. Exposure to cigarette smoke, allergies among family members and daycare attendance proved to be important factors in inhaler use frequency.

**Conclusions:** Postnatal environmental factors are important in predicting and modifying early childhood outcomes among preterm infants.

## Introduction

Bronchopulmonary dysplasia (BPD) is one of the most prevalent and significant morbidities among preterm infants (1–3). In modern neonatology, BPD refers to arrested lung development and its origin is considered to be multifactorial (4, 5). Preterm infants who develop BPD have been shown to be at increased risk for chronic respiratory morbidity, cardiovascular impairment, and neurodevelopmental delay (3, 6). The diagnostic criteria for BPD have changed over the years to reflect contemporary neonatal care (7–10). Yet despite improvements in neonatal care, BPD rates have not declined and evidence-based therapies for BPD prevention are still scarce (11–13). Multiple environmental exposure factors have been suggested as potential contributors to the progression or resolution of lung disease and to developmental outcomes among preterm infants, among them factors associated with air pollution, nutrition, postnatal infections and genetics. Yet the current literature is limited regarding the impact of post-discharge environmental factors on respiratory and developmental outcomes among preterm infants with BPD (14). Understanding these interactions may help improve the respiratory and developmental outcomes of these infants.

The present study was conducted among a recently born cohort of preterm infants cared for at a single tertiary medical center. We aimed to determine the effect of BPD severity on developmental and respiratory outcomes during the first 2 years of life and to examine the contribution of a wide range of perinatal and postnatal factors to these outcomes. A better understanding of these factors will enable us to individualize discharge and follow-up plans for these infants.

## Methods

### Patient Selection

Infants born prior to 32 weeks gestational age (GA) between January 2012 and August 2015 and also completed a follow-up exam at the preterm follow-up clinic at 1-year corrected age (CA) were eligible for the study. Excluded were infants with major congenital anomalies and higher order multiples. Parents were retrospectively contacted (between 2016 and 2018) and asked to complete a health and lifestyle questionnaire investigating their child's first 2 years of life. The final cohort included only those who had completed a follow-up exam at 1-year corrected age and completed the health and lifestyle questionnaire.

### Definitions

#### Maternal and Neonatal Variables

Maternal and neonatal variables were obtained from the electronic medical records. Severe brain injury was defined as intraventricular hemorrhage (IVH) grade ≥3 or periventricular leukomalacia. Necrotizing enterocolitis (NEC) was defined as Bell classification stage ≥2. Severe ROP (ROP) was defined as any ROP that was treated. The severity-based definition of BPD as defined by the NIH was used (9). NIH classifies BPD as mild, moderate or severe according to the amount of supplemental oxygen and the mode of respiratory support administered at 36 weeks GA among infants requiring supplemental oxygen at 28 days. In order to improve the study's statistical power, infants with moderate and severe BPD were combined into one group, so that the study groups were: no BPD, mild BPD and moderate-severe BPD.

#### Health and Lifestyle Questionnaire

Mothers who agreed to participate were asked to complete a questionnaire assessing their infant's past medical history, respiratory health and lifestyle, along with that of other family members (Supplementary Material 1). Mothers had the option to complete the questionnaire during their visit to the follow-up clinic, by phone or via e-mail.

#### Developmental Outcomes

Infants born prior to 32 weeks GA are followed up in the developmental clinic at 10–14 months corrected age. Griffith's Mental Development Scales (GMDS) were administered by a trained neurodevelopmental neonatologist. The GMDS yield scores on five subscales: locomotor, personal-social, hearing and language, eye and hand coordination, and performance. The GMDS generate a raw score that is converted into a developmental quotient The mean score for each subscale is 100 and the standard deviation is 15 (15). We decided to restrict the study to infants born between 2012 and 2015, since a new GMDS version was launched in 2016, thus increasing the risk of bias.

#### Respiratory Outcomes

The following respiratory outcomes were recorded: any hospitalizations, respiratory-related hospitalizations, pulmonologist visits, use of systemic steroids, inhalation use (bronchodilators or steroids), and frequency. Three levels of bronchodilator use were defined: (1) none or rare; (2) intermediate (>6 courses/year); and (3) frequent (daily treatment for ≥3 months/year).

#### Statistical Analysis

The data were analyzed using Python version 3.8. Statistical significance was established at *p* < 0.05. Participants with >20% missing data were eliminated from the analysis, while those with only a few missing observations were imputed with the average. For the second analysis, we removed observations with no GMDS scores. Baseline maternal and infant characteristics and respiratory outcomes were compared across the three groups (no BPD, mild BPD, and moderate to severe BPD). Differentiation was assessed using the Chi-square test for proportions and the Mann-Whitney test for averages. The difference in GMDS between the BPD severity groups was assessed using a non-parametric Kruskal-Wallis test. The association between GMDS at 12 months CA and severity of BPD was assessed using a linear regression model. Models were regulated using ridge and lasso regularization as well as support vectors to avoid overfitting in this small sample size. The prediction performances of the models were evaluated using a leave-one-out cross-validation scheme by computing the mean absolute error (MAE) averaged across participants. A recursive feature elimination (RFE) was applied in combination with the models in order to select the best subset of features.

The association between respiratory outcomes and BPD severity was assessed using a Chi-square test for proportions. For significant Kruskal-Wallis outcomes, we applied a Mann-Whitney test between groups. A machine-learning-based classification model was used to determine factors that may influence frequency of inhaler use. In order to fit an algorithm that achieved the highest accuracy for the data set with the lowest possibility of overfitting, we examined the following algorithms in a grid search analysis: Random Forest, logistic regression classifier, gradient boosting classifier, XGBoost and ExtraTree classifier (Supplementary Figure 1). The XGBoost classifier proved to be the best machine-learning algorithm with the highest weighted accuracy (f1 score) and the lowest difference between the train and test metric, indicating the smallest probability of overfitting.

## Results

### Study Population

**Figure 1:**
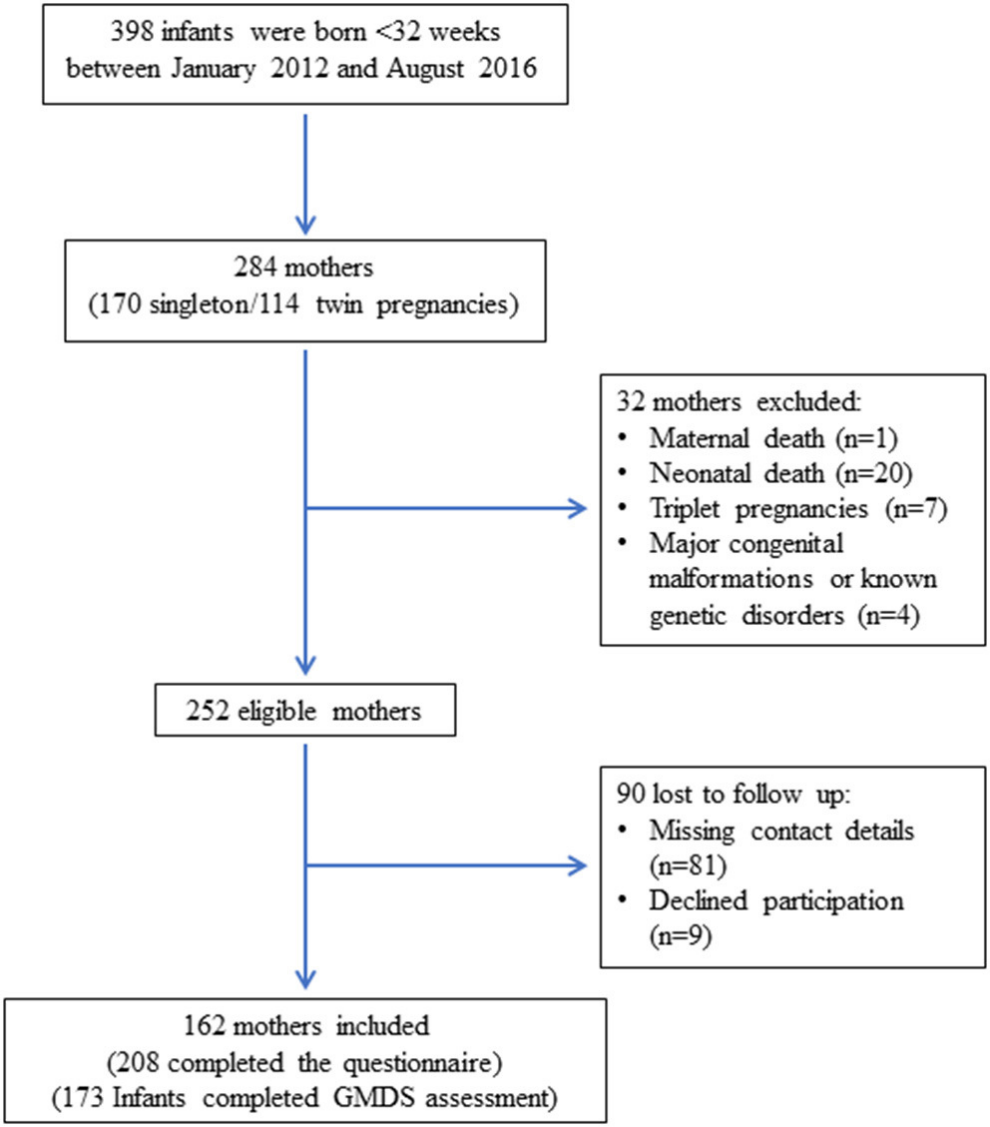
Flow chart of the study cohort.

During the study period, 284 mothers gave birth to 398 infants born at <32 weeks' gestation. Thirty-two mothers (11.3%) were excluded due to the following: maternal death (*n* = 1), neonatal death (*n* = 20), triplets (*n* = 7), genetic abnormalities or major congenital malformations (*n* = 4). Of the 252 eligible mothers, 162 (64.2%) mothers of 208 infants completed the questionnaire. Of the 90 mothers (35.7%) that did not respond, 81 could not be reached and nine declined to participate. Of the 208 infants included, 173 (83.2%) had GMDS assessment at 12 months CA and completed questionnaires (Figure 1).

### Baseline Characteristics

Demographic and clinical characteristics are shown in Table 1. Of the 208 infants included, 152 (73.1%) were not diagnosed with BPD, 40 (19.2%) had mild BPD and 16 (7.7%) had moderate-severe BPD. Infants diagnosed with moderate-severe BPD were born to mothers with significantly fewer years of education and were significantly more likely be male. Those in the no-BPD group were born later (30.5 ± 1.3 vs. 27.5 ± 1.5 vs. 28 ± 2.3 weeks, *p* < 0.001) and had a higher BW (1,351 ± 290 vs. 994 ± 224 vs. 994 ± 339 g, *p* < 0.001) compared to those in the other groups, respectively. The duration of invasive mechanical ventilation was directly associated with BPD severity. Those in the moderate-severe group required fewer days to regain birth weight. Infants with no-BPD were significantly less likely to be diagnosed with sepsis, severe brain injury or ROP compared to the other two groups. Necrotizing enterocolitis occurred in 12.5% of those with moderate to severe BPD, compared with 2.5% among the mild-BPD group and 1.3% in the no-BPD group (*p* < 0.05). Parental allergies were significantly less common among the mild BPD group and were comparable for the no-BPD and moderate-severe BPD groups. Exposure to cigarette smoke occurred in 50% of those with moderate-severe disease, compared to 25% of the no-BPD group and 12.5% of the mild BPD group (*p* < 0.005). Ninety percent of the no-BPD children attended daycare, compared to 69% of the moderate-severe group.

**Table 1:** Maternal, neonatal, and environmental characteristics.

**Descriptive characteristics**	**No BPD** **(*n* = 152)**	**Mild BPD** **(*n* = 40)**	**Moderate-severe BPD** **(*n* = 16)**	***P*-value**
**Maternal characteristics**				
Maternal age (years)	32.4 (5.9)	31.5 (6.2)	30.5 (6.3)	NS
Maternal education (years)	15 (0.05)	15.2 (2.8)	14 (3.5)	0.004^a^^,^^c^
**Income level**				
High	49 (32.2%)	13 (32.5%)	4 (25.0%)	NS
Medium	59 (38.8%)	15 (37.5%)	3 (18.7)	
Low	44 (29.0)	12 (30.0%)	9 (56.3%)	
Maternal hypertension	19.7%	7.5%	18.75%	NS
Chorioamnionitis	12 (7.9%)	4 (10%)	3 (18.75%)	NS
Surgical delivery	51 (33.5%)	14 (35/0%)	5 (31.3%)	NS
**Neonatal characteristics**				
Gestational age (weeks)	30.5 (1.3)	27.5 (1.5)	28 (2.3)	<0.001^a^^,^^b^
Birth weight (grams)	1,351 (290)	994 (224)	994 (339.5)	<0.001^a^^,^^b^
Female	84 (55.2%)	22 (55.0%)	4 (25%)	<0.05^a^^,^^c^
Exposure to breastmilk	122 (80.3%)	33 (82.5%)	11 (68.8) 11	NS
**Clinical characteristics**				
Duration of invasive mechanical ventilation (days)	0.8 ± 1.6	4.7 ± 5.9	12.9 ± 14.1	<0.01^a, b, c^
Days to regain birth weight	13 ± 5.1	16.4 ± 6.8	11.4 ± 6.8	<0.001^b^^,^^c^
Sepsis	23 (15.1%)	16 (42%)	6 (37.5%)	<0.05^a^^,^^b^
Severe brain injury	22 (14.5%)	20 (50%)	6 (37.5%)	<0.05^a^^,^^b^
Necrotizing enterocolitis	2 (1.3%)	1 (2.5%)	2 (12.5%)	<0.05^a^
Treated ROP	0%	1 (2.5%)	2 (12.5%)	<0.05^a^^,^^b^
**Environmental risk factors**				
Asthma/allergy (mother)	24 (15.8 %)	1 (2.5%)	5 (31.2%)	<0.05^b^^,^^c^
Asthma/allergy (father)	35 (23.0%)	2 (5.0%)	3(18.7%)	<0.05^b^
Cigarette smoking parent	38 (25.0%)	5 (12.5%)	8 (50%)	<0.05^a^^,^^c^
RSV immunization	145 (95.4%)	40 (100%)	16 (100%)	NS
Day care attendance	137 (90.1%)	31 (77.5%)	11 (69%)	<0.05^a^^,^^b^
Exposure to animals	33 (21.7%)	13 (32.5%)	3 (18.8 %)	NS

*BPD, bronchopulmonary dysplasia; ROP, retinopathy of prematurity; RSV, Human Respiratory Syncytial Virus*.

*Continuous variable expressed as mean and SD. Binary variables expressed as rate*.

*Results of multiple comparison analysis*.

a *Significant differences between No BPD and Moderate-Severe BPD*.

b *Significant differences between No BPD and Mild BPD*.

c *Significant difference between Mild BPD and Moderate-Severe BPD*.

### Developmental Outcomes

**Figure 2:**
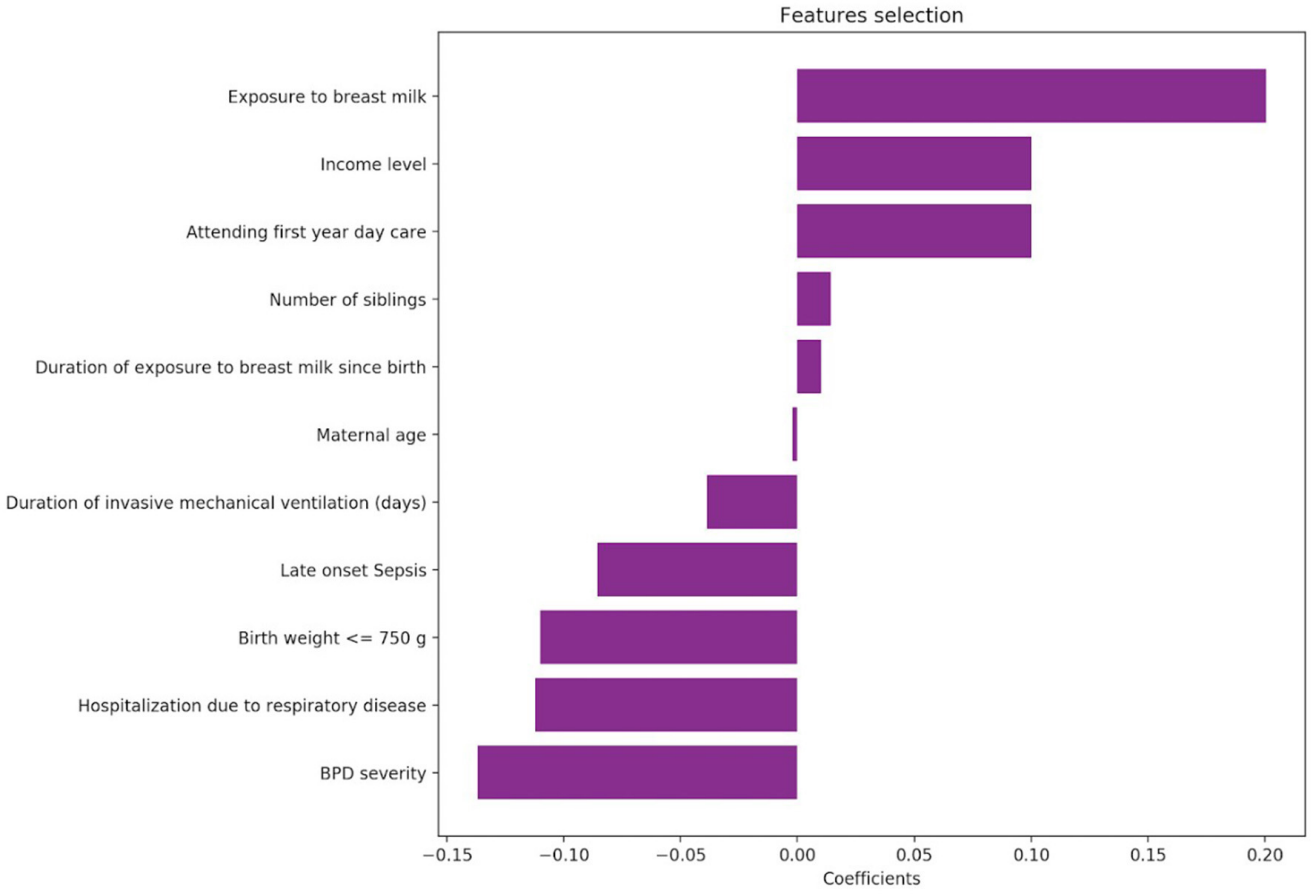
Factors that had an impact on the GMDS.

A total of 173 infants completed the GMDS assessment at 12 months CA. Table 2 illustrates the GMDS scores according to BPD severity groups. We found that moderate-severe BPD was associated with a significantly lower developmental score on all subscales except for the personal-social scale. The linear regression model demonstrated that the following factors had a positive impact on GMDS scores: exposure to breastmilk, income level, daycare attendance, siblings, duration of breastfeeding, younger maternal age. Factors that negatively affected GMDS scores included BPD severity, hospitalization due to respiratory complications, birth weight of <750 g, sepsis and duration of mechanical ventilation (Figure 2).

**Table 2:** Developmental outcomes.

	**No BPD** **(*n* = 127)**	**Mild BPD** **(*n* = 33)**	**Moderate-Severe BPD** **(*n* = 13)**	***P*-value**
Gross motor	97.7 (12.8)	95.8 (14.2)	86.6 (13.4)	0.0016^a^^,^^b^
Social emotional	108 (14.1)	108.3 (13.1)	95.6 (23)	0.076
Speech and language	90.7 (13.6)	88 (13.3)	73.8 (14.4)	0.0004^a^^,^^b^
Hand eye coordination	101.3 (16.2)	99.7 (14.9)	86.2 (18.8)	0.0067^a^^,^^b^
Performance	99.3 (10.9)	97.8 (11.7)	85.2 (16.4)	0.007^a^^,^^b^
Total score	99.3 (10.9)	97.8 (11.7)	85.2 (16.4)	0.0008^a^^,^^b^

*BPD, bronchopulmonary dysplasia*.

*Results of multiple comparison analysis*.

a *Significant differences between No BPD and Moderate-Severe BPD*.

b *Significant differences between Mild BPD and Moderate-Severe BPD*.

### Respiratory Outcomes

**Figure 3:**
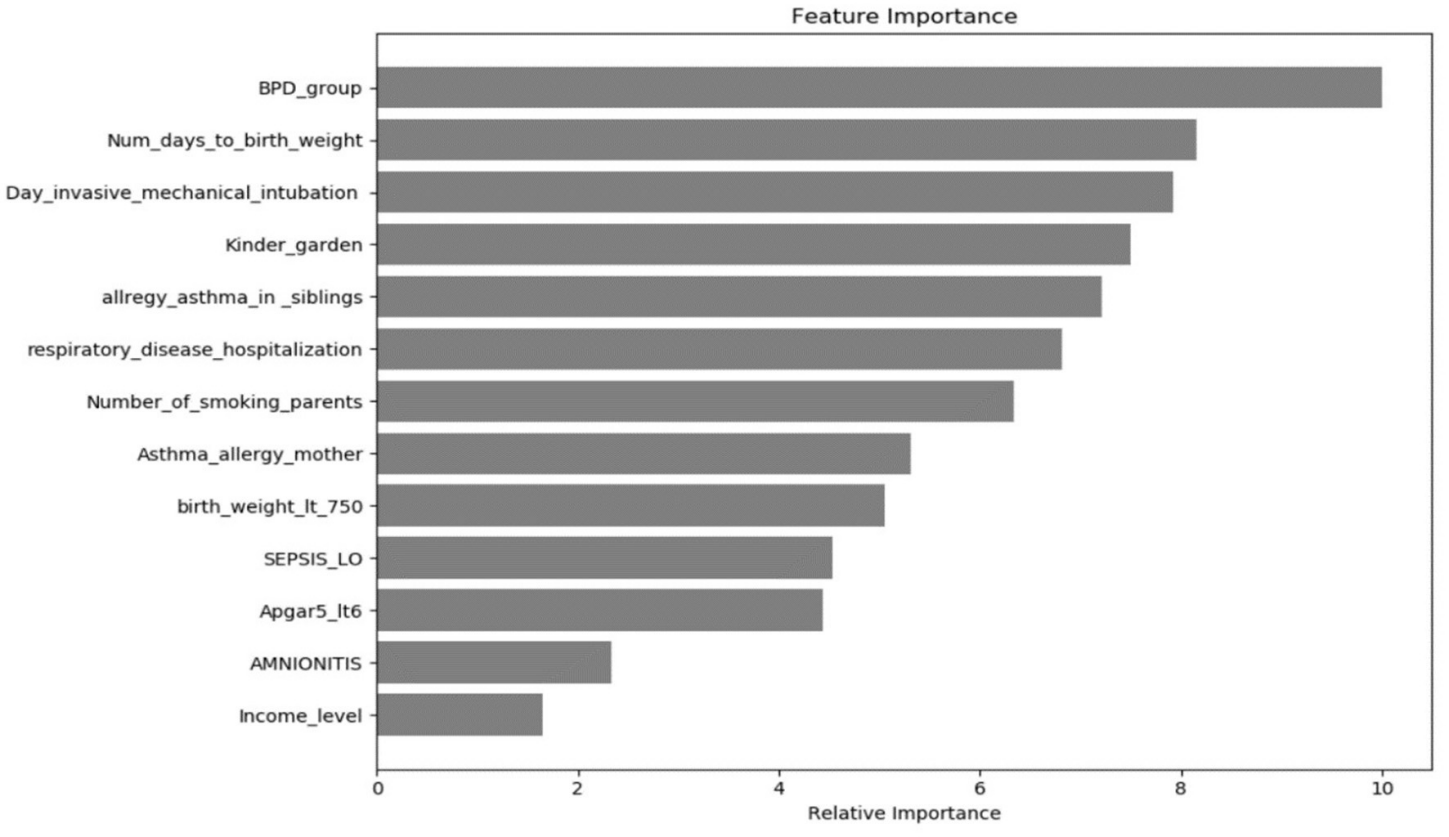
Factors that proved to be important and affected the use of inhaler.

None of the included infants required respiratory support at >12 months CA. A comparable percentage of infants in all groups were re-hospitalized for respiratory as well as non-respiratory indications. Pulmonologist follow-up correlated with BPD severity. The groups did not differ in their exposure to systemic steroids or inhalations. Frequency of inhaler use differed significantly between the no-BPD, mild BPD, and moderate-severe BPD groups (Table 3). Figure 3 shows the factors affecting inhalation use according to their significance. BPD severity proved to be the most significant prognostic factor, followed by days to regain birth weight and duration of mechanical ventilation.

**Table 3:** Respiratory outcomes.

	**No BPD** **(*n* = 127)**	**Mild BPD** **(*n* = 33)**	**Moderate -Severe BPD** **(*n* = 13)**	
Any hospitalization	25 (19.6%)	7 (21.2%)	3(23.0%)	NS
Respiratory illness related hospitalization	21 (16.5%)	7 (21.2%)	3(23.0%)	NS
Any systemic steroids	38 (29.9%)	14(42.4%)	5 (38.4%)	NS
Inhaler use (yes)	65 (66.9%)	20(60.6%)	9 (69.2%)	NS
**Inhaler frequency**
None	70 (55.1%)	21 (63.6%)	5 (38.4%)	*P* = 0.0014
Intermediate	51 (40.1%)	7 (24.2%)	4 (30.8%)	
Frequent use	6 (4.7%)	5 (15.1%)	4 (30.8%)	
Pulmonologist visit	12 (9.4%)	11(33.3%)	8 (61.5%)	<0.001^a^^,^^b^

*BPD, bronchopulmonary dysplasia; NS, not significant*.

*Results of multiple comparison analysis*.

a *Significant differences between No BPD and Moderate-Severe BPD*.

b *Significant differences between No BPD and Mild BPD*.

## Discussion

The present study aimed to identify factors that may impair or ameliorate early childhood developmental and respiratory outcomes among a cohort of preterm infants at a single medical center diagnosed with BPD. Comparison analysis confirmed the findings of previous literature by demonstrating that BPD severity is associated with various perinatal factors, among them gestational age, birth weight, gender, duration of invasive mechanical ventilation and time to regain birthweight (11).

In the second step we examined the association between BPD severity and early childhood outcomes (neurodevelopmental and respiratory). The results indicated that moderate-severe BPD is associated with a significantly greater risk for lower developmental scores at 12 months CA and with increased frequency of inhaler use during the first 2 years of life. In the third step we looked for possible environmental factors that may play a role in modifying these outcomes. In addition to perinatal factors that are already known, the results showed that exposure to breast milk and early daycare attendance have a positive impact on developmental scores, while exposure to cigarette smoke and family members with asthma are important factors in determining respiratory disease burden. The inclusion of various postnatal environmental factors as modifiers reflects real life and may also suggest new options for risk assessment and early interventions. The results of this study also support previous findings by showing that those with moderate-severe disease are at significant higher risk than those in the two other groups. Moreover, those with mild disease were shown to have similar outcomes as those with no BPD, thus reinforcing the need for new criteria to determine BPD and eliminating oxygen need at 28 days of life as a criterion.

In current study, preterm infants with no or mild BPD had comparable developmental scores, while infants with moderate-severe BPD had significantly lower developmental scores. Moreover, the developmental scores of infants with moderate-severe BPD were one standard deviation (SD) below the normal population. Although developmental assessment later in life (>18–24 month corrected age) provides a better prediction of long-term outcomes, early identification of infants at risk may facilitate earlier intervention. Multiple cohort studies and randomized control trials have reported an increased risk for neurodevelopmental delay among preterm infants with BPD (16–19). Jensen et al. recently reported that the level of respiratory support at 36 weeks postmenstrual age accurately predicted death as well as respiratory and neurodevelopmental impairment at 18–26 months CA (10). Lodha et al. used BPD severity-based criteria to study outcomes. They reported that neurodevelopmental disability, and particularly cognitive impairment at 36 months CA, was significantly more frequent in children with moderate-severe BPD (20). Our results support the use of severity-based criteria in identifying those at risk for unfavorable outcomes.

To date, no one factor or combination of factors has been shown to reliably predict which infants with BPD will experience neurodevelopmental delays (21). According to the findings of the current study, outcomes are influenced by environmental and familial factors in addition to other perinatal factors and BPD severity. Factors associated with a favorable neurodevelopmental outcome include breastmilk exposure and duration, income level, early daycare attendance, having siblings, and maternal age. Factors found to be associated with unfavorable developmental outcomes included birth weight <750 g, sepsis, duration of mechanical ventilation and re-hospitalization due to respiratory etiology. These results shed light on the importance of post-discharge factors in modifying developmental outcomes. Our data show that infants with moderate-severe disease were less likely to attend daycare. Caregivers should be made aware of the importance of peer interaction to early childhood development, and parents should be encouraged to support this interaction, even in medically vulnerable populations (22).

In terms of early respiratory disease burden, 20–25% of all the children in the study were re-hospitalized at least once, regardless of BPD severity. Those with moderate-severe disease were significantly more likely to use inhalers frequently and have pulmonologist follow-ups.

We suggest that since a comparable number of children in all three groups used inhalers, the more frequent use among the moderate-severe group is a true reflection of their disease burden. Moreover, it is possible that the higher rate of pulmonologist follow-ups among those with more severe disease generated a positive modification of the results of respiratory disease burden. This finding is in line with previous reports that preterm infants referred to a pulmonologist at discharge had a lower rate of emergency room visits and re-hospitalizations (23).

Previous studies have reported that early childhood wheezing is associated with antenatal maternal smoking, atopy, male sex, breastfeeding and other exposure factors (24, 25). In the present study, we used machine-learning algorithms to assess the relative significance of various factors on inhaler use. Machine-learning algorithms make it possible to analyze a small data base, which otherwise would not be possible. In this analysis, BPD severity was found to be the most influential factor, followed by duration of mechanical ventilation. Environmental factors associated with increased inhaler use were daycare attendance, exposure to secondary smoke inhalation, asthma among family members and exposure to breastmilk. Many of these factors were previously reported to be associated with respiratory outcomes among children born prematurely (26–30). Perinatal and developmental data were collected in real time, the parents completed the questionnaire retrospectively, such that their answers may be subject to inaccuracies. To minimize inaccuracies, we avoided asking for details such as number of hospitalizations. Second, we assessed inhalation frequency rather than using the gold standard of lung function tests. While inhaler prescription varies among caregivers, inhaler use may better reflect respiratory disease burden. The third limitation is the small number of infants. Despite combining two groups, the numbers were small. We tried to overcome this problem by using machine-learning statistical methods. Machine-learning, a branch of artificial intelligence, uses data and algorithms to imitate the way humans learn, such that analysis accuracy gradually improves. In machine-learning the analysis learns from the data and identifies factors that are important for the searched outcomes. Machine-learning trains algorithms to generate a prediction model, which is then optimized by adjustment to known examples to improve accuracy. This study may also be prone to recall bias as all members of the cohort, and particularly the sickest ones, did not attend the follow-up clinic or respond to the questionnaire. Despite these limitations, this is an important analysis that adds to the growing body of literature on this topic.

The strengths of this study include the large cohort of preterm infants who were followed up and the large body of data available for each infant, including perinatal, postnatal, and post-discharge data. This comprehensive list of variables is more relevant to real life. These results may be particularly important for directing resources to the most vulnerable at-risk group.

In summary, this is one of few studies to investigate the importance of environmental factors in modifying early childhood morbidities among preterm infants diagnosed with BPD. Although this study describes the experience of a single medical center, thus limiting generalization of its findings, we demonstrate that factors such as nutrition, daycare attendance, and cigarette smoking and asthma among family members play an important role in modifying outcomes. Physicians should consider these factors when counseling families and planning follow-ups.

## Data Availability Statement

The raw data supporting the conclusions of this article will be made available by the authors, without undue reservation.

## Ethics Statement

The studies involving human participants were reviewed and approved by Sheba Medical Center. Written informed consent from the participants' legal guardian/next of kin was not required to participate in this study in accordance with the national legislation and the institutional requirements.

## Author Contributions

IM conceptualized and designed the study, coordinated and supervised data collection, analyzed the data and drafted the initial manuscript, and reviewed and revised the manuscript. EB conducted the statistical analysis and reviewed and revised the manuscript. YW and OS-C coordinated and supervised data collection, co-analyzed the data, and revised the manuscript. AE and OL co-analyzed the data and revised the manuscript. SP-K helped review the literature and drafted and reviewed the manuscript. NO analyzed the data, drafted the initial manuscript, and reviewed and revised the manuscript. All authors approved the final manuscript as submitted and agreed to be accountable for all aspects of the work.

## Conflict of Interest

The authors declare that the research was conducted in the absence of any commercial or financial relationships that could be construed as a potential conflict of interest.

## Publisher's Note

All claims expressed in this article are solely those of the authors and do not necessarily represent those of their affiliated organizations, or those of the publisher, the editors and the reviewers. Any product that may be evaluated in this article, or claim that may be made by its manufacturer, is not guaranteed or endorsed by the publisher.
