# Supplementary material for: Predictors of Developmental and Respiratory Outcomes Among Preterm Infants With Bronchopulmonary Dysplasia
Source: Front Pediatr. 2021 Nov 25;9:780518. doi: 10.3389/fped.2021.780518 (PMC8667805; doi:10.3389/fped.2021.780518)
Supplement: Supplementary file 1 [file Data_Sheet_1.docx]

Questionnaire for family

Date of completion First Name Last Name I.D. No.

Please mark the right answer

1. Is this child a twin?
2. If yes, is this child the first or the second twin?
3. Was your child immunized against RSV during the first year of life?
4. Does your child receive routine immunizations?
5. Has your child been re-hospitalized since being discharged from the NICU?
6. If your answer to question 5 is yes, please indicate at what age……………, where……….. and for what diagnosis……………
7. Does your child have any allergies ……………….? Does your child have allergic nasal discharge ………………….? If so, please indicate the allergen to which the child is sensitive…………………….? What are the child’s symptoms ………………………?
8. Has your child ever been treated with an inhaler? yes/ no
9. If you answered yes to question 8, please indicate the type of inhaler? What medication was given? Beta-mimetic? Steroids? Saline? How frequently?
10. Has your child ever been treated with systemic steroids? if yes, please indicate the type of steroids and the duration? What was the reason for this treatment?
11. Has your child been routinely seen by a pulmonologist?
12. Has your child been diagnosed with any chronic condition? If yes, please give details…………
13. Has your child been seen by OT/PT?
14. At what age did child begin attending a nursery/ kindergarten (with more than two other children)?
15. In what type of educational setting is your child currently enrolled?
16. Is there any history of allergies/asthma in the family? ………mother?.................father………..? siblings………….?
17. Smoking: mother y/n? father yes/no?
18. Do you have any pets at home………….? If yes, what kind?
19. Was your child fed breast milk?
20. If yes, for how long? Did your child receive breast milk exclusively?
21. Was your child fed from the breast?
22. At what age was your child weaned, and why?
