# Supplementary material for: Predictors of Developmental and Respiratory Outcomes Among Preterm Infants With Bronchopulmonary Dysplasia
Source: Front Pediatr. 2021 Nov 25;9:780518. doi: 10.3389/fped.2021.780518 (PMC8667805; doi:10.3389/fped.2021.780518)
Supplement: Supplementary file 2 [file Data_Sheet_2.docx]

Fig . Machine learning Analysis

Supplementary figure 1. Machine learning search analysis


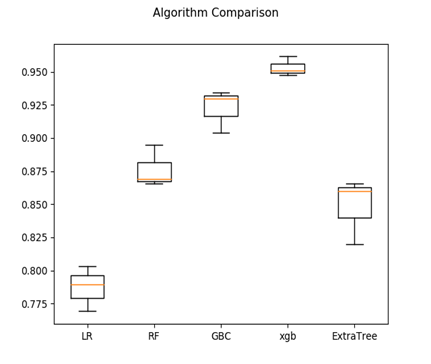


Random forests (RF), Logistic regression classifier (LR), Gradient boosting classifier (GBC), XGBoost (xgb), and ExtraTree classifier (ExtraTree).
